# Lived experience and family engagement in psychiatry research: A scoping review of reviews

**DOI:** 10.1111/hex.14057

**Published:** 2024-04-28

**Authors:** Lisa D. Hawke, Natasha Y. Sheikhan, Terri Rodak

**Affiliations:** ^1^ University of Toronto Toronto Canada; ^2^ Centre for Addiction and Mental Health Toronto Canada

**Keywords:** family engagement, lived experience, mental health, patient and public involvement, patient engagement, patient‐oriented research, psychiatry

## Abstract

**Background:**

A growing body of research is addressing the process and science of engaging people with lived experience (PWLE) of mental health challenges and other psychiatric conditions, and family members, in research activities.

**Objective:**

This scoping review of reviews synthesizes literature reviews on the engagement of PWLE and family members in research across the field of psychiatry.

**Method:**

Systematic searches were conducted in seven bibliographic databases. Records were independently screened first at the title and abstract level, then at the full‐text level. Included were any literature synthesis studies published in English, French, or Spanish in any given year, focusing on the engagement of PWLE and/or family members in research within psychiatry. Twenty records were included. Data were extracted in a spreadsheet and codebook thematic analysis was used across the body of articles to synthesize the findings.

**Results:**

Aspects of PWLE engagement have been synthesized in 20 review articles reviewing 376 articles across psychiatry as a whole and several subpopulations, including youth mental health, dementia, neurodevelopmental disorders, people who use drugs, and forensic mental health. Information specific to family engagement is lacking. Barriers, facilitators, and positive impacts of PWLE engagement have been widely reported across domains of research, with a considerable degree of consensus across subpopulations. Some negative impacts and reporting challenges have also been identified.

**Discussion:**

This scoping review of reviews provides an overarching understanding of the current state of the science of PWLE and family engagement across psychiatry research. The findings can inform future research practices enriched with a genuine and effective engagement with PWLE and families.

**Patient or Public Contribution:**

The authorship team includes members with intersecting lived experience and academic identities. Additional lived experience engagement was not conducted as part of this review.

## 1 BACKGROUND

A growing body of research is addressing the process and science of engaging people with lived experience (PWLE) of mental health challenges, addictions, and other psychiatric conditions, as well as family members (/F), in research endeavours.^1^ This type of approach is also known as patient‐oriented research, patient engagement in research, and patient and public involvement,^2^, ^3^ although PWLE is the preferred terminology for many of these partners.^4^ Family members or informal caregivers are generally included under the umbrella term ‘patient’,^2^ with the goal of providing input into the experience of supporting and caring for a loved one with these challenges or conditions.

There are a variety of motivations for PWLE/F engagement in research. From an integrated knowledge translation (iKT) perspective, a range of stakeholders should be engaged in all stages of a research project to increase research relevance and uptake, with engagement included from project conceptualization to knowledge mobilization and beyond.^5^ Among the key partners to include in iKT are PWLE/F, who have important insights to share.^6^ Engagement can also be considered an anti‐oppressive practice given the historical and ongoing experience of inequities in health research and service provision,^7^ particularly within the field of psychiatry. While PWLE/F engagement is increasingly seen across the health sciences, this includes a growing emphasis on engagement in the field of psychiatry across a wide range of populations, study designs, and research questions.^1^, ^8^, ^9^

Engagement in psychiatry research follows many of the same principles as engagement in other spheres of health research, yet there are several unique factors that should be taken into consideration in this field. Notably, injustices and oppression specific to mental health,^7^ high levels of stigma,^10^, ^11^, ^12^, ^13^ and resulting mistrust in research institutions can pose particular barriers to engagement.^1^, ^14^ Trauma‐informed engagement is a high priority,^15^ given high rates of past trauma among individuals with mental health challenges. While psychiatry includes a heterogeneous group of health challenges, representativeness and inclusion are particularly important to productive and authentic engagement practices. In addition, the intersections between complex family dynamics and supportive families who want to contribute to care and research add challenges to family engagement in psychiatry.^16^ The nature of some psychiatric conditions may affect the way individuals choose to and are able to engage. Research frameworks, theories, and processes specific to PWLE/F engagement in psychiatry research are therefore required.

The scientific literature examining aspects of engagement practices, experiences and outcomes is rapidly expanding. Several literature synthesis studies have emerged, addressing different aspects of engagement or different subpopulations.^1^, ^17^ As the literature accumulates, there is a need to synthesize the findings in a comprehensive manner to gain a high level of understanding of this work across the various domains of psychiatry, for example with various subpopulations. This review emerges from a single large tertiary care hospital with a substantial engagement infrastructure in place traversing the entire field of psychiatry and the subpopulations within it; the importance of synthesizing the literature across the discipline emerges from our experience with operationalizing engagement across subpopulations within psychiatry, including across conditions and age groups of interest. Synthesizing these findings will support the development of an understanding of aspects of engagement practices that can guide the psychiatry research community.

## 2 OBJECTIVE

This scoping review of reviews synthesizes the literature reviews conducted on the engagement of PWLE/F in psychiatry research.

## 3 METHODS

We conducted an overview of reviews using the scoping review methodology given the breadth and nature of the research, using established scoping review processes.^18^, ^19^ We chose the scoping review methodology to map the literature on engagement.^20^ This methodology is appropriate as we comprehensively mapped the breadth of the evidence in the current literature, identified thematic areas, and synthesized the evidence. We ensured rigour by following an established scoping review methodology consisting of five major steps for the review:^21^ (1) defining the research question, (2) identifying relevant studies, (3) screening and selecting studies, (4) extracting the data, and (5) summarizing the data and reporting. Extracted data were analysed using a codebook approach to thematic analysis. We conducted and reported on the review in compliance with the PRISMA Extension for Scoping Reviews (PRISMA‐ScR) guidelines.^22^ The review protocol was not published or registered.

### 3.1 Defining the research question

This review aimed to map the academic literature reviewing PWLE/F engagement in psychiatry research. Using the PCC framework (population, concept, context), this review addresses PWLE of health challenges within the domain of psychiatry (population), or family members of these individuals, engaged in academic research within psychiatry (inclusive of mental health and substance use challenges, behavioural addictions, neurodevelopmental conditions, etc.) as partners, collaborators, advisors, or other roles beyond participants (concept), as described in academic literature review articles of any design (context).^23^

### 3.2 Identifying relevant studies

A health sciences librarian (T.R.) developed a comprehensive search strategy, then conducted the searches. The search was initially designed, tested, and finalized in APA PsycInfo, and then adapted for and conducted in the following additional bibliographic databases: Ovid MEDLINE, Embase, Cumulative Index to Nursing & Allied Health Literature (CINAHL), Scopus, Applied Social Sciences Index and Abstracts (ASSIA), and Cochrane Central Register of Controlled Trials (CENTRAL). The strategy queried two concepts using database‐specific subject headings, natural language keywords, and advanced search operators. One cluster aimed to capture items about PWLE/F engagement in research through terms such as ‘co‐creation,’ ‘expert by experience’, and patient/family/lived experience terms paired with participation/engagement and research terms via an adjacency operator. The other cluster aimed to capture all items within the sphere of behavioural health including psychiatry, mental health, trauma, substance use, behaviour disorders, neurodivergence, and neurology—all of which may fall under the umbrella of psychiatry research. These terms and clusters were combined using Boolean operators, and then limited to literature reviews using database‐specific limits and keywords describing review types. The searches were limited to review articles (any design), but no search limitations were placed on publication year or language, to ensure an inclusive search. A hand search of Google Scholar was also conducted, as well as a search of the reference lists of the identified reviews. Given that the review aimed to collect literature on engagement within academic research, we limited the grey literature search to dissertations in APA PsycInfo and nontraditional publications in the CINAHL database. The final core search strategy for APA PsycInfo is available as supplementary material.

### 3.3 Study screening and selection

Inclusion and exclusion criteria are listed in Table 1. To be included, articles had to be literature synthesis studies of any design that synthesized literature on the engagement of PWLE/F in academic research, in psychiatry as a whole or specific to any subfield of psychiatry. Articles could synthesize the literature on any subpopulation within this scope. They could be included if published in English, French, or Spanish, although only English‐language publications were identified. We excluded any articles in other languages, or that did not present findings from literature synthesis studies, as well as any that conceptualized ‘engagement’ as involvement in activities other than research (i.e., engagement in clinical services, engagement in service design outside of research frameworks, engagement in policymaking).

**Table 1:** Inclusion and exclusion criteria.

Inclusion criteria	Exclusion criteria
Literature synthesis study (any design)	Primary research or opinion papers
Published in English, French, or Spanish	Engagement outside of psychiatry
Any time period	Engages other stakeholders, not PWLE or families
Focus on the engagement of PWLE or families in research (inclusive of design, conduct, data analysis, interpretation, and knowledge translation)	Engagement is not in research processes
Research is in psychiatry	

Abbreviation: PWLE, people with lived experience of mental health and/or substance use challenges.

**Figure 1:**
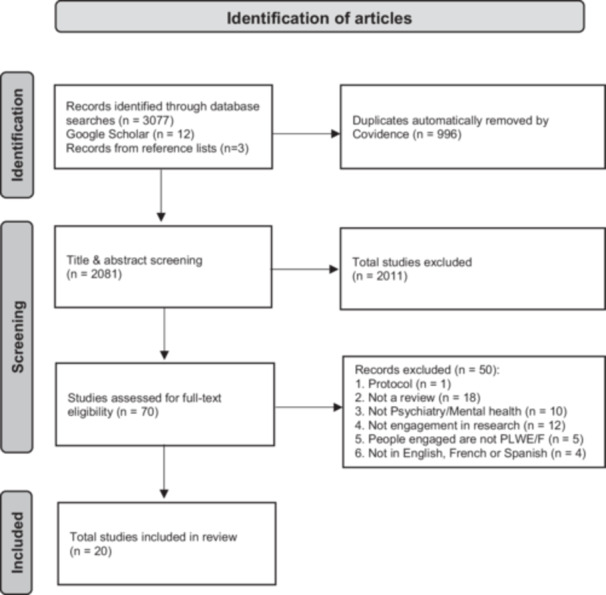
PRISMA flowchart for documents identified in the scoping review.

All identified records were uploaded into Covidence,^24^ an online platform for systematic review management. Duplicates were identified and removed by the automatic duplicate detection system, with manual removal of any duplicates identified thereafter. Three independent reviewers screened all remaining records first by title and abstract (two reviewers per abstract). Any disagreements at this stage were discussed and resolved by consensus together with the research lead. The remaining records were screened at the full‐text level by the same three reviewers, with discussion and consensus‐based decision‐making until a final set of included articles was established. A PRISMA diagram was developed to represent the search results (Figure 1).

### 3.4 Data extraction and analysis

A data extraction tool was developed in a spreadsheet software to extract basic study identifying information (authors, year of publication, journal, country) and descriptive information (study objective, synthesis design, population, methodology, family engagement). Data extraction was conducted by one team member and cross‐checked by a second member. The articles were then uploaded into NVivo 12^25^ for codebook thematic analysis.^26^ All articles were subjected to thematic analysis to synthesize key messages put forth in the articles, regardless of the methodology followed by the individual article. The research team worked together to develop a codebook based on a preliminary understanding of the literature to answer the research aims. One team member then conducted the coding, with iterative refinements of codes and frequent discussions and refinement with the research lead. The analysis focused on identifying content in relation to the research question.

### 3.5 Data summarization and reporting

Study identifiers and descriptive data were summarized in table format. Topics were summarized in narrative format. We did not conduct a quality assessment, given that scoping reviews typically do not include this process and the literature is heterogeneous in nature.^27^

## 4 RESULTS

### 4.1 Overview

A total of 20 records were found that met the inclusion criteria (Figure 1). The final set of included reviews is described in Table 2. It includes scoping reviews (*n* = 10), systematic reviews (*n* = 3), other types of reviews (*n* = 4), and unspecified review methodologies (*n* = 3). The total number of unique articles reviewed by the 20 included articles after removing duplicates was 376.

**Table 2:** Summary of included review articles.

References	Methodology	Population	Years included	Languages included	Term describing engagement	Papers reviewed (*N*)	Objective	Family/carer inclusion
Ali et al.^17^	Scoping review	Youth mental health	2005 to 2021	English	Patient engagement	16	Review youth engagement approaches; synthesize barriers and facilitators and mitigating strategies; provide recommendations for youth engagement in research	No carer‐specific objective No carer‐specific search terms Reports on carer engagement in included studies Minimal mention of carer roles or implications
Bethell et al.^28^	Scoping review	Dementia	Through to 2018	English, French	Patient engagement	54	Synthesize the approaches, barriers, enablers and impacts of patient engagement in dementia research.	Carer‐specific objective Carer‐specific search terms Reports on carer engagement in included studies Discusses carer roles or implications
Brierley‐Jones et al.^29^	Not specified	Acute mental health care users	From 2000	Not specified	Patient involvement	52	Synthesize patient‐engaged research focusing on improving safety in acute mental healthcare settings	No carer‐specific objective Carer‐specific search terms Reports on carer engagement in included studies Does not discuss carer roles or implications
Burton et al.^30^	Not specified	Dementia	2018 to 2019	English	Patient and public involvement (PPI)	16	Update a previous scoping review on patient and public involvement in dementia research, focusing on methods, barriers, and effective strategies	No carer‐specific objective No carer‐specific search terms Reports on carer engagement in included studies Minimal mention of carer roles or implications
Di Lorito et al.^31^	Not specified	Dementia, older adults, learning disabilities, and mental health service users	Not specified	Any	Peer researchers	7	Synthesize the literature on peer researchers in dementia research, focusing on effective strategies, risks, benefits, and practical aspects to propose positive practices.	No carer‐specific objective No carer‐specific search terms Does not report on carer engagement in any included studies Minimal mention of carer roles or implications
Happell et al.^32^	Integrative review	People with mental health challenges and physical health concerns	2000 to 2015	English	Participatory mental health consumer research	4	Synthesize the nature and the scope of the evidence on the engagement of people with mental illness in research on physical health care for individuals with mental illness	No carer‐specific objective No carer‐specific search terms Reports on carer engagement in included studies Does not discuss carer roles or implications
Hawke et al.^33^	Scoping review	People with mental health challenges	2017 to 2022	English	Engagement of people with lived experience	49	Examine the gaps in the evidence on and implementation of engagement in mental health and substance use research	No carer‐specific objective No carer‐specific search terms Does not report on carer engagement in any included studies Does not discuss carer roles or implications
Jennings et al.^34^	Critical literature review	People with mental health challenges	From 2007	Not specified	Patient and public involvement (PPI)	10	Propose, pilot, and refine a methodology for coresearcher involvement in qualitative data analysis and suggest a corresponding best practice framework	No carer‐specific objective No carer‐specific search terms Does not report on carer engagement in any included studies Does not discuss carer roles or implications
Jivraj et al.^35^	Scoping review	Autism and neurodevelopmental conditions	Through 2013	English	Participatory research	7	Describe participatory research partnerships in research on autism spectrum disorders or other neurodevelopmental disorders and synthesize the impact on research	No carer‐specific objective No carer‐specific search terms Reports on carer engagement in included studies Does not discuss carer roles or implications
Kowe et al.^36^	Systematic review	Dementia or mild cognitive impairment	2000 to 2020	English	Participatory research	9	Synthesize the literature on the positive and negative impacts of participatory dementia research.	No carer‐specific objective No carer‐specific search terms Reports on carer engagement in included studies Does not discuss carer roles or implications
Martins^37^	Systematic review	Youth mental health	2000 to 2020	Not specified	Patient and public involvement (PPI)	18	Synthesize the impacts, enablers, and evaluation methods in youth‐focused patient and public involvement in mental health research	No carer‐specific objective No carer‐specific search terms Reports on youth carer engagement in included studies Does not discuss carer roles or implications
McCabe et al.^38^	Systematic review	Youth mental health	2000 to 2022	Not specified	Youth engagement	16	Synthesize the evidence on the impacts, barriers, and facilitators of engagement in youth mental health research and propose recommendations.	No carer‐specific objective Carer‐specific search terms Reports on carer engagement in included studies Minimal mention of carer roles or implications
Miah et al.^39^	Scoping review	Dementia	2000 to 2018	Any	Patient and public involvement (PPI)	20	Synthesize methods for and impacts of patient and public involvement in dementia research in the European Union.	No carer‐specific objective No carer‐specific search terms Reports on carer engagement in included studies Does not discuss carer roles or implications
Sangill et al.^9^	Scoping review	People with mental health challenges	Through 2017	Danish, English, Norwegian, Swedish	Collaborative research	32	Synthesize the research on the engagement of mental health service users in research.	No carer‐specific objective No carer‐specific search terms Reports on carer engagement in included studies Does not discuss carer roles or implications
Semrau et al.^40^	Narrative review	People with mental health challenges	Through 2013	English, Spanish, Portuguese, French, German	Service user and caregiver involvement	20	Synthesize the literature on patient and caregiver engagement in mental health policy, service, and research work in lower and middle income countries and identify best practices for evaluating engagement.	Carer‐specific objective No carer‐specific search terms Reports on carer engagement in included studies Minimal mention of carer roles or implications
Sheikhan et al.^1^	Scoping review	People with mental health challenges	2012 to 2022	English	Engagement of people with lived experience	61	Synthesize the literature on the impacts, barriers, and facilitators for patient engagement in mental health and substance use research.	No carer‐specific objective No carer‐specific search terms Does not report on carer engagement in included studies Does not discuss carer roles or implications
Souleymanov et al.^41^	Scoping review	People who use drugs	1985 to 2013	English	Community‐based participatory research	25	Summarize the evidence on ethical issues and promising practices regarding community‐based participatory research among people who use drugs.	No carer‐specific objective No carer‐specific search terms Does not report on carer engagement in included studies Does not discuss carer roles or implications
Völlm et al.^42^	Rapid review	Forensic	1980 to 2016	English	Involving or engaging service users in research	23	Synthesize the literature on engaging people using forensic mental health services in research and make recommendations for this area of work.	No carer‐specific objective Carer‐specific search terms Does not report on carer engagement in included studies Does not discuss carer roles or implications
Wang et al.^43^	Scoping review	Dementia	Through 2018	English	Involving people with dementia in design research	26	Synthesize the literature on the impacts of engaging people with dementia to co‐design research, the evolution of this approach over time, and tools, limitations, and recommendations for research co‐design with this population	No carer‐specific objective No carer‐specific search terms Reports on carer engagement in included studies Discusses carer roles or implications
Zhang and Ying^44^	Scoping review	People with mental health challenges	Through 2018	Not specified	Participatory action research	7	Synthesize the literature on the application of participatory research in psychiatry.	No carer‐specific objective No carer‐specific search terms Reports on carer engagement in included studies Does not discuss carer roles or implications

The included review articles covered a breadth of areas of psychiatry, including general psychiatric populations as well as specific populations. Three articles focused on engagement in youth mental health research.^17^, ^37^, ^38^ Another six focused on engagement in dementia research.^28^, ^30^, ^31^, ^36^, ^39^, ^43^ Other specific populations included people with autism or neurodevelopmental disorders,^35^ people who use drugs,^41^ people in forensic mental health services,^42^ and general mental health in acute care settings.^29^ Several articles focused on mental health research in general.^1^, ^9^, ^32^, ^33^, ^34^, ^40^, ^45^ One review sought articles specific to PWLE/F engagement in mental health research, specifically in low or middle‐income countries, but did not find any.^40^

Information specific to the engagement of family members was largely lacking (Table 2). Only two reviews had family or carer‐specific objectives and only four included family or carer‐specific search terms. Despite this, 13 of 20 reviews reported that at least some aspect of family engagement in the articles reviewed, although most did not pursue any substantial discussion of the implications of family engagement.

While the reviews had a variety of populations and specific objectives (Table 2), they generally addressed three primary topic areas: (1) barriers to engagement, (2) facilitators of engagement, and (3) impacts of engagement. Reporting issues were discussed in nine reviews. Findings from these topics are synthesized below.

### 4.2 Barriers to engagement

Many of the reviews discussed the barriers to effective and authentic engagement of PWLE in research (see Table 3). Most barriers were reported across multiple populations. Barriers included challenges related to equity, diversity, inclusion, and accessibility (EDIA); challenges with relationships and communication; logistical and operational barriers; barriers specifically related to the mental health of the individuals engaged; and power‐related barriers. EDIA‐related barriers included factors such as diversity, representativeness, language and literacy, and jargon‐related issues. Relationship and communication barriers included difficulties understanding and managing roles and expectations, as well as challenges navigating conflicting perspectives and tensions. A lack of strong, reciprocal communication and ongoing feedback was also highlighted, as well as the sense of being disconnected or disengaged. Limited and nonreciprocal communication was seen as hindering the research environment. Logistical barriers included factors such as geographical location, scheduling and attendance, time, and funding requirements. Operational barriers included a lack of early and sustained engagement, along with recruitment challenges, insufficient planning, a complex research culture, and challenges navigating research ethics boards and administrative requirements, sometimes in the context of a lack of research experience, skills, and knowledge of engagement on the part of participants and a lack of engagement knowledge by researchers. Some reviews pointed to barriers specifically related to the mental health of the individuals engaged, such as anxiety with regard to speaking in a group or distress about the research topic at hand. The symptoms of dementia can also be a barrier to effective engagement specifically in the dementia research sphere. Barriers related to relational power dynamics manifested in the form of unbalanced research hierarchies and resistance to change, tokenism, and stigma, alongside mistrust, fears, or negative views of research.

**Table 3:** Barriers to engagement across sub‐populations of psychiatric research.

Categories	Subcategories	General MH	Dementia	Youth	Autism	PWUD	Forensic	References
Challenges related to EDIA	Difficulty with diversity, representativeness, and engaging hard to reach groups	X	X	X				[1, 17, 28, 30, 31, 33]
	Impact of long term engagement on representation		X	X			X	[30, 38, 42]
	Jargon and technical language	X	X	X			X	[1, 9, 31, 37, 42]
	Language and literacy barriers	X	X				X	[1, 30, 42, 43]
Challenges related to relationships and communication	Difficulties with expectations, e.g., managing expectations, unmet expectations	X	X	X			X	[9, 17, 30, 38, 42]
	Feeling disconnected, unsupported, or disengaged	X	X					[1, 30]
	Lack of communication, feedback, or reciprocity	X		X		X		[1, 9, 37, 41]
	Navigating diverse and conflicting perspectives and tensions	X		X			X	[1, 38, 42]
Logistical barriers	Geographic location	X		X				[1, 17]
	Scheduling and attendance issues	X	X			X		[1, 30, 41]
	Time and funding constraints	X	X	X				[1, 17, 28, 30, 31, 36, 38, 43]
Operational barriers	Lack of early involvement and sustaining engagement	X		X	X			[1, 34, 35, 38]
	Lack of research experience and learning curve	X	X					[1, 9, 28, 30]
	Nature of research culture and process (complex, competitive)	X	X					[1, 28]
	Navigating ethics boards and administrative barriers	X	X	X		X	X	[1, 28, 38, 41, 42]
	Recruitment		X	X			X	[17, 30, 38, 42]
	Researchers’ limited knowledge on engagement	X						[1]
Challenges related to mental health	Challenges related to symptoms of dementia		X					[28, 30, 31]
	Discomfort, anxiety, and potential for distress	X	X			X		[1, 9, 28, 30, 41]
Power‐related barriers	Mistrust, fears, or negative views of research	X				X	X	[1, 41, 42]
	Power imbalances and research hierarchies	X	X	X			X	[1, 9, 17, 30, 31, 38, 42]
	Resistance to change and adapting to sharing control	X	X					[1, 28]
	Stigma and labelling	X		X		X		[1, 17, 34, 41]
	Tokenism	X					X	[1, 9, 42]

Abbreviations: EDIA, equity, diversity, inclusion and accessibility; MH, mental health; PWUD, people who use drugs.

### 4.3 Facilitators of engagement

The facilitators of engagement are described in detail in Table 4. Again, a wide range of facilitators were reported across multiple domains of psychiatry research. These revolved around inclusive practices, clear planning of authentic engagement, and aspects of roles and relationships, which reflected the identified barriers. Inclusivity involved using accessible language; clear, flexible, and ongoing communication; and being reflexive and well‐informed. Accounting for the individual characteristics of the PWLE was considered important to inclusivity, such as by providing appropriate accommodations, opportunities, and privacy, while holding engagement activities in a safe, familiar space, with refreshments, employing an anti‐oppressive and trauma‐informed lens, and accounting for the needs and strengths of those engaged. A further facilitator was to provide clear, early, realistic plans for appropriate ongoing engagement and specifying all members’ roles and responsibilities. Planning needsincluded training, as well as the time and funding required, such as fair compensation and incentives for PWLE. It is important to invest resources in relationship building and engagement oversight, including attempts to balance power dynamics and avoiding tokenistic engagement. Through strong relationships, PWLE should be able to access opportunities to authentically participate in decision‐making and to be acknowledged for their contributions.

**Table 4:** Facilitators to engagement across dimensions of psychiatric research.

		General MH	Dementia	Youth mental health	Autism	PWUD	Forensic	References
Categories	Subcategories							
Inclusive practices: Accessible language and clear communication	Accessible and inclusive reading materials and communication tools	X	X	X	X		X	[1, 30, 34, 35, 38, 42, 43]
	Clear and jargon‐free communication	X	X	X			X	[1, 9, 28, 31, 33, 37, 38, 42]
	Communicating impact and incorporating feedback of PWLE	X	X	X		X		[1, 30, 37, 38, 41]
	Flexibility and patience	X	X	X				[1, 28, 30, 31, 37, 38, 43]
	Incorporating nonverbal language and visual prompts	X	X					[30, 31, 34, 43]
	Ongoing updates and feedback		X	X			X	[28, 30, 38, 42]
	Pre‐ and de‐briefing	X	X	X				[1, 29, 31, 33, 38]
	Remaining reflexive and well informed	X	X	X		X		[1, 34, 38, 41, 43]
	Sensitivity around terms and labels	X					X	[32, 33, 42]
Inclusive practices: Accounting to the individual characteristics of PWLE	Accommodations, e.g., extra time, breaks, doing daily activities		X	X			X	[30, 38, 42, 43]
	Accounting for individual strengths, skills, needs, and capacity	X	X			X		[1, 28, 30, 31, 34, 41, 43]
	Anti‐oppressive and trauma‐informed lens	X		X				[1, 33, 38]
	Attending to socio‐cultural nuances, norms, and needs					X	X	[41, 42]
	Ensuring privacy and confidentiality					X	X	[41, 42]
	Familiar and informal environments, especially outside of MHSU setting	X	X	X			X	[1, 17, 30, 31, 34, 37, 38, 42, 43]
	Having a range of supports available, e.g., emotional support, institutional support	X	X	X	X	X	X	[1, 9, 17, 28, 31, 33, 34, 35, 38, 41, 42]
Planning for authentic engagement	Clearly defined roles, responsibilities, and expectations	X	X	X			X	[1, 9, 28, 30, 31, 34, 38, 42]
	Early planning and engagement	X	X	X			X	[1, 28, 33, 34, 38, 42]
	Engagement throughout the research process	X		X	X			[1, 9, 32, 34, 35, 37]
	Recruiting diverse PWLE voices	X	X	X		X	X	[1, 17, 30, 33, 34, 38, 41, 42]
	Realistic planning and goal‐setting	X	X					[31, 33, 34]
	Time and funding	X	X	X		X	X	[1, 28, 30, 31, 33, 34, 36, 37, 38, 39, 41, 42]
	Training and mentorship for PWLE	X	X	X		X	X	[1, 9, 28, 30, 31, 33, 34, 36, 37, 38, 41, 42]
Roles and relationships	Having an engagement coordinator or liaison	X		X			X	[1, 38, 42]
	Investing in relationship‐building	X	X	X		X	X	[1, 9, 28, 29, 30, 31, 33, 34, 36, 37, 38, 41, 42, 43]
	Opportunities for authentic contributions	X	X			X		[1, 9, 28, 34, 41, 44]
	Treated as equals and equalizing power dynamics	X	X	X	X	X	X	[1, 9, 31, 33, 34, 35, 36, 37, 38, 39, 41, 42]

Abbreviations: MH, mental health; MHSU, mental health and substance use; PWLE, people with lived experience; PWUD, people who use drugs.

### 4.4 Impacts of engagement

The impacts of engagement were discussed in detail by many reviews, across domains of research, and are described in Table 5. Reviews identified impacts of engagement on research outputs and outcomes, PWLE, researchers, study participants, and the community. Extensive impacts were reported on the research outputs, notably in terms of acceptability and relevance, team dynamics, methodology and design, knowledge translation, and overall an enhanced authenticity, credibility, trustworthiness, quality, validity, integrity, richness, and rigour. Many articles described the strong impact engagement had across all of the reviewed studies. Impacts on PWLE included increased aspects of empowerment, improved social connectedness, and professional and personal development. Impacts on researchers included improved communication and planning skills, new engagement networks, and shifts in perspectives and understandings; researchers further gained a sense of pride, motivation, and accountability. Engagement was considered to bring added value to study participants through better rapport and trust, enhanced safety and comfort, increased disclosure, and was considered to bring an added value to participants as a whole. Impacts on the community included stigma reduction, improvements to services, policies and practices, and ultimately an improved public awareness and perceptions of research.

**Table 5:** Impact of engagement across dimensions of psychiatric research.

		General MH	Dementia	Youth mental health	Autism	PWUD	Forensic	References
Categories	Subcategories							
Impact on research outputs and outcomes	Improved methodological aspects	X	X	X	X	X		[1, 30, 32, 35, 36, 37, 38]
	Improved team dynamics	X	X	X		X		[1, 31, 36, 37, 38, 39, 41]
	Increased relevance and acceptability of the research to PWLE	X	X	X	X	X		[1, 31, 32, 35, 37, 38, 39, 41, 44]
	Seen as helpful and valuable to the research process	X	X		X			[1, 9, 34, 35, 36]
	Improved knowledge translation and dissemination	X	X	X	X			[1, 9, 17, 28, 29, 30, 31, 32, 34, 35, 36, 37, 38, 39, 41, 44]
Impact on PWLE	Improved facets of empowerment	X	X	X	X	X	X	[1, 9, 30, 31, 35, 37, 38, 41, 42]
	Improved social connectedness and reduced isolation	X	X	X		X		[1, 30, 31, 38, 41]
	Professional and personal development	X		X	X	X	X	[1, 9, 35, 37, 38, 41, 42]
Impact on researchers	Built communication and planning skills		X	X				[36, 37]
	Built engagement networks		X	X				[30, 38]
	Shift in perspective and understanding	X	X	X				[1, 9, 31, 36, 37, 38]
Impact on study participants	Built rapport and trust with participants	X		X				[1, 9, 37]
	Enhanced safety and comfort of participants	X		X				[1, 9, 38]
	Increased level of disclosure from participants			X	X			[35, 37]
	Positive difference and added value to participants	X	X				X	[1, 31, 42]
Impact on the community	Stigma reduction	X	X			X		[1, 30, 31, 41]
	Improvements to services, policies, and practice	X	X	X		X		[1, 29, 37, 39, 41]
	Increased public awareness and improved perceptions of research		X	X				[30, 37]
Negative impacts	Negative emotional or interpersonal impact	X	X			X	X	[9, 31, 36, 41, 42, 43]
	Negative impact on methods and rigour		X	X			X	[36, 38, 42]
	Negative or null impact on process	X	X	X				[1, 9, 36, 38, 43]

Abbreviations: MH, mental health; PWLE, people with lived experience; PWUD, people who use drugs.

However, some negative impacts were identified, which were also consistent across domains of research. At the emotional and interpersonal level, reviews pointed out the burden or stress on PWLE or families, and uncomfortable emotions such as feeling unvalued, inferior, or frustrated. These were usually in response to the identified barriers to engagement, such as challenges with communication, feedback, and unresolved expectations. In terms of the research process, negative impacts further reflect the barriers to engagement, with highlighted impacts including the additional time, effort, resources, and responsibility needed, failure to balance power dynamics, and more challenging decision‐making. Negative or null impacts of engagement on recruitment were also noted in some reviews.

### 4.5 Reporting of engagement

Nine reviews described challenges with regard to the reporting of PWLE/F engagement. Inadequate reporting was considered a hindrance to understanding the depth of the engagement conducted, assessing its quality, and understanding its impact.^1^, ^28^, ^29^, ^35^, ^37^ Notably, some reviews highlighted inconsistencies with regard to defining engagement and selecting terminology to describe PWLE/F, reporting factors that enabled or hindered engagement and its impact, and describing engagement processes.^1^, ^17^, ^35^, ^37^ Reviews noted that the role PLWE/F played in the reporting process was unclear; for example, it was not always possible to determine whether any of the co‐authors were in PWLE/F roles or to understand their representativeness in the absence of reporting of sociodemographic characteristics.^1^, ^41^, ^42^ In light of these challenges, there was a perceived need to standardize the reporting of engagement and engagement terminology, increase the uptake of reporting guidelines, and increase the use of robust measurements for impact.^1^, ^17^, ^28^, ^29^, ^33^

## 5 DISCUSSION

The literature on PWLE/F engagement in psychiatry research has been advancing rapidly in recent years. Our scoping review of reviews synthesizes understandings of PWLE/F engagement across psychiatry research, by mapping review articles published across psychiatry as a whole, together with sub‐fields and sub‐populations, to support the implementation of engagement across the breadth of psychiatry research. A range of reviews examined PWLE/F engagement. The primary areas of focus of these reviews were the barriers, facilitators, and impacts of engagement. Within these areas of discussion, considerable overlap was found across domains of psychiatry. Indeed, findings regarding barriers, facilitators and impacts were highly consistent across general mental health, dementia, and youth mental health, which have been substantially reviewed. Results focused on diversity and inclusive practice; communication, relationships and power; logistics and daily operations and planning. These are consistent with some of the gaps in the science of engagement recently identified.^46^ A notable gap across the breadth of the literature was the engagement of families or caregivers in research, which is insufficiently attended to across general mental health and subpopulations. Challenges with regard to consistent and appropriate reporting of PWLE/F engagement are also worthy of note.

Engagement has been synthesized in general mental health research or psychiatry researchand in a number of subpopulations of psychiatry research. The literature has been extensively synthesized, with consistent results across mental health populations in general, as well as youth mental health and dementia. Some work has also been conducted on autism or neurodevelopment, substance use, and forensics, although results were less consistent since fewer reviews have been conducted, generating fewer insights. Indeed, the literature synthesis work on these areas of engagement was sparser, leaving some gaps in our understanding of engagement with these populations. For example, the impact of engagement in the forensic sphere has yet to be thoroughly synthesized. In the case of areas unreported upon, it is unclear whether these are specific gaps in the state of the literature, as opposed to barriers and facilitators that do not emerge in that area of research; however, given that most of the findings are consistent across at least two population groups, with many emerging across multiple domains when more literature syntheses are available, similar impacts might be hypothesized to emerge in the areas not yet reported upon. These are areas for future research to bolster the state of the literature.

While the populations are diverse, this is not an exhaustive list of areas of psychiatry, leaving some additional gaps. Researchers conducting PWLE/F‐engaged research in other populations are encouraged to consider how engagement practices might work in their domains of research, document their findings, and continue expanding the knowledge base, with particular attention to domain‐specific issues. For example, mental health‐related factors were found to be a barrier to engagement in some subpopulations,^9^, ^28^, ^31^ pointing to the need to synthesize the findings as they are available across a wider range of specific diagnostic groups and domains of research, such as depression, psychotic spectrum disorders, and other areas of work.^47^, ^48^ While challenges with memory might be expected to be specific to engagement in dementia or related research, challenges with regard to generating distress might be present across a more extensive set of subpopulations. Mitigating strategies, then, are needed for specific populations and more broadly for the discipline as a whole. Engagement in lower and middle‐income countries was an additional identified gap that merits further work.^40^

Barriers and facilitators of engagement have been extensively reported on in a wide variety of reviews across subpopulations of psychiatry, with considerable consistency across subpopulations. This is important as it suggests that they have been identified and synthesized to a sufficient degree to systematically inform engagement models, frameworks, and practices for the discipline moving forward. Researchers embarking upon or further developing their engagement initiatives are encouraged to take these barriers and facilitators into consideration and explicitly develop mitigating strategies that enable them to improve the quality of their engagement work. Researchers might consider focusing on ensuring inclusive practices, strong planning, and attention to authentic relationships to optimize the quality of their engagement, while implementing specific mitigating strategies to address barriers. A next important step to advance the science of engagement is for engagement‐focused research teams to report directly on the mitigating strategies that they have employed successfully—or unsuccessfully—to address the barriers identified across the literature and in their own work, to help advance engagement practices in a pragmatic manner.

There is a consensus across the literature that PWLE/F engagement has a wide range of positive impacts on PWLE/F, researchers, and research projects, with benefits extending to the community at large. A number of negative impacts were also noted; however, these negative impacts aligned substantially with the barriers to engagement, which in turn aligned with the facilitators. For example, a negative impact is the time it takes to engage,^36^, ^38^, ^43^ while a barrier is not having enough time^28^ and a facilitator is having enough time.^37^ Time, then, emerges as a key factor that requires attention, planning, and mitigating strategies to support strong engagement practices. Likewise, negative relational and associated emotional impacts were identified,^31^, ^36^ which align with relational barriers and facilitators. Future research is therefore needed to understand how to optimize relationships within complex engagement dynamics. Concrete solutions to barriers and optimization of facilitators can minimize negative impacts and ensure that engagement is a positive experience and beneficial process.

An area in which a synthesis was largely lacking was the engagement of family members in psychiatry research. While patient‐oriented research frameworks consider family members under the concept of ‘patient’,^2^ family engagement had been searched for and reported on inconsistently in the literature and its specific implications were minimally discussed. This is unfortunate, given that family members have direct experience in many aspects of the mental health and care experience and can provide valuable input.^49^ Family engagement has specific benefits, but also entails specific challenges in this area of work,^16^ perhaps differentially in different psychiatric subpopulations. Researchers are called on to consider the unique characteristics of family engagement and to advance discussions on addressing the inherent challenges while harnessing the inherent benefits of it.

Limited diversity of engagement was noted by a number of reviews and is a known challenge with PWLE/F engagement.^50^, ^51^ In understanding engagement, it is important to understand who is engaged and who they represent. However, in the context of PWLE/F as colleagues rather than research participants, alongside disclosure concerns and confidentiality rights, data on their sociodemographic profiles is not generally collected or reported. This leaves gaps in the understanding of diversity, intersectionality, and representativeness of engagement, including intersecting factors such as cultural or ethnic background, sex and gender, diagnostic groups, symptomatic severity, and level and discipline of education, which can include advanced degrees within or outside of psychiatry and mental health. Future work is required to understand the minimum and optimal reporting recommendations needed to understand and address diversity limitations in the context of privacy, ethics, and disclosure considerations.

Indeed, clear and consistent reporting of engagement methods and impacts was revealed as a challenge across PWLE/F engagement in psychiatry research. Challenges in reporting include inconsistent and nontransparent reporting that make it difficult to understand how engagement was conducted, with whom, and to what degree. Poor reporting poses a challenge to reproducing reliable and high‐quality evidence. Reporting guidelines are available to guide many aspects of health research, such as randomized‐controlled trials,^52^ systematic reviews,^53^ economic evaluations,^54^ and qualitative research.^55^ Reporting guidelines for patient engagement in research are available,^56^ but these are not specific to mental health, are subject to criticism,^57^ and appear to be under‐utilized.^58^ Future research is required to develop best practices in reporting PWLE/F engagement in psychiatry research, with attention to developing or adapting reporting guidelines that will meet the needs of PWLE/F and researchers alike.

This review is the most comprehensive literature review of PWLE/F engagement in psychiatric research to date, to our knowledge. By synthesizing the findings from across psychiatry as a whole, it provides incremental learnings about the state of the science and practice of PWLE/F engagement in this area of research. However, it is subject to a number of limitations. Since the focus is on synthesizing reviews of engagement in psychiatry, newly emerging primary research on the topic is not represented and may have important emerging findings. Likewise, reviews conducted on health and social care research outside of the domain of psychiatry were not included but may have relevant findings. Reviews published in languages other than English, French and Spanish were not included, for pragmatic reasons. While articles in French and Spanish could be included if identified, the search was not translated and specifically conducted in these languages, and no such articles were identified. Although a broad range of bibliographic databases were searched, it is possible that reviews may exist that are not indexed in the databases employed for this review and that were therefore missed. A substantial grey literature search was not conducted; however, since this review is limited to literature synthesis studies, we consider that this is not problematic as these studies are predominantly found in the peer‐reviewed literature.

## 6 CONCLUSIONS

PWLE/F engagement is being conducted and results synthesized across subpopulations within psychiatry, with discussions focusing on a range of common barriers, facilitators, and impacts. The consistency observed across populations suggests the level of evidence is sufficient to systematically inform engagement models, frameworks, and practices. However, family or carer engagement remains an under‐investigated area of work. Likewise, increasing diversity and ensuring rigorous reporting of engagement require future work, as both have potentially critical impacts on ensuring and understanding representativeness across domains of research, accounting for personal characteristics and intersectionalities. Nevertheless, PWLE/F engagement appears to be a relevant practice across a broad range of subpopulations within psychiatry research, as well as psychiatry research as a whole, with considerable positive impacts for PWLE/F, researchers, research projects, study participants, and communities.

## AUTHOR CONTRIBUTIONS

**Lisa D. Hawke**: Conceptualization; methodology; formal analysis; supervision; project administration; resources; writing—original draft; writing—review and editing; data curation; funding acquisition; validation; software. **Natasha Y. Sheikhan**: Methodology; formal analysis; writing—review and editing; writing—original draft; writing—review and editing; validation. **Terri Rodak**: Methodology; investigation; writing—review and editing; data curation.

## CONFLICT OF INTEREST STATEMENT

The authors declare no conflict of interest.

## Supporting information

Supporting information.

## Data Availability

The final search strategy has been published as supplementary material to the final review and all data is presented herein.
