# Supplementary material for: Lived experience and family engagement in psychiatry research: A scoping review of reviews
Source: Health Expect. 2024 Apr 28;27(3):e14057. doi: 10.1111/hex.14057 (PMC11056206; doi:10.1111/hex.14057)
Supplement: Supplementary file 1 — Supporting information. [file HEX-27-e14057-s001.docx]

| APA PsycInfo <1806 to July Week 4 2023> | | |
| --- | --- | --- |
| 1 | exp mental disorders/ | 1031436 |
| 2 | exp psychiatry/ | 57027 |
| 3 | (mental* or psychiatr* or neuro*).ti,id,hw. | 855510 |
| 4 | (post-trauma* or posttrauma* or PTSD or complex trauma or developmental trauma or CPTSD).ti,ab,id,hw. | 66128 |
| 5 | ((disorder* adj2 eating) or anorex* or bulimi*).ti,ab,id,hw. | 45323 |
| 6 | (suicide* or selfharm* or self-harm* or selfinjur* or self-injur*).ti,ab,id,hw. | 73864 |
| 7 | ((behavio?r* or gambl* or shop* or buy* or spend* or steal* or theft*) adj3 (disorder* or addiction* or compuls* or problem* or pathological*)).ti,ab,id,hw. | 108226 |
| 8 | (Kleptomania* or Trichotillomania or (hair adj3 pull*) or dermatillomania or excoriation or (skin adj3 pick*)).ti,ab,id,hw. | 2116 |
| 9 | (neurodiver* or neuro-diver* or attention deficit* or ADHD or autism or autistic or asperger* or ASD or tourette*).ti,ab,id,hw. | 110366 |
| 10 | Emotion* dysregulation.ti,ab,id,hw. | 4203 |
| 11 | Oppositional defian*.ti,ab,id,hw. | 4076 |
| 12 | (Conduct adj3 (disorder* or problem* or issue*)).ti,ab,id,hw. | 13290 |
| 13 | (Behavio?r* adj3 (disorder* or problem* or disrupt*)).ti,ab,id,hw. | 98721 |
| 14 | (externaliz* or externalis* or internalize* or internalis*).ti,ab,id,hw. | 33848 |
| 15 | (dementia* or alzheimer*).ti,ab,id,hw. | 119925 |
| 16 | ((substance* or drug* or tobacco or nicotine or alcohol* or cannabis or marijuana or stimulant* or steroid* or analgesic or sedative* or cocaine or hallucinogen* or psilocybin or amphetamine* or methamphetamine* or benzodiazepine* or opioid* or opiate* or heroin or fentanyl or inhalant* or depressant* or barbiturate*) adj3 ("use" or user* or misus* or abus* or disorder* or depend* or addict* or withdraw* or detox* or overdose* or recovery)).ti,ab,id,hw. | 268125 |
| 17 | SUD.ti,ab,id,hw. | 4833 |
| 18 | ("People who use drugs" or PWUD).ti,ab,id,hw. | 869 |
| 19 | ((assist* or supervis* or manag* or treat* or inpatient* or residential*) adj3 (withdraw* or detox*)).ti,ab,id,hw. | 4658 |
| 20 | ((opioid* or opiate*) adj3 (replacement or substitution or maintenance)).ti,ab,id,hw. | 1725 |
| 21 | (opioid agonist* adj3 (treatment* or therap*)).ti,ab,id,hw. | 715 |
| 22 | ((medica* assisted or opioid assisted) adj3 (treatment* or therap*)).ti,ab,id,hw. | 824 |
| 23 | (naloxone or methodone or buprenorphine or suboxone or sublocade).ti,ab,id,hw. | 9353 |
| 24 | or/1-23 | 2150953 |
| 25 | client participation/ | 3172 |
| 26 | (co-produc* or coproduc*).ti,ab,id. | 1793 |
| 27 | (co-design* or codesign*).ti,ab,id. | 1152 |
| 28 | (co-creat* or cocreat*).ti,ab,id. | 3998 |
| 29 | (co-construct or coconstruct*).ti,ab,id. | 950 |
| 30 | (collaborat* adj2 methodol*).ti,ab,id. | 194 |
| 31 | ("patient* and public* involvement" or "public* and patient* involvement").ti,ab,id. | 497 |
| 32 | advisory group*.ti,ab,id. | 517 |
| 33 | expert* by experience*.ti,ab,id. | 223 |
| 34 | peer researcher*.ti,ab,id. | 133 |
| 35 | ((patient* or client* or "service user*" or consumer* or survivor* or informant* or family or families or carer* or caregiver* or consumer* or child* or youth or young people) adj2 (participat* or engag* or advisor* or partner* or involv* or collaborat*) adj5 research*).ti,ab. | 3352 |
| 36 | ((patient* or client* or "service user*" or consumer* or survivor* or informant* or family or families or carer* or caregiver* or consumer* or child* or youth or young people) adj2 (participat* or engag* or advisor* or partner* or involv* or collaborat*)).ti,ab,id. and research*.id,hw. | 3394 |
| 37 | (("lived expertise" or "living expertise" or "lived experience" or "living experience" or PWLE*) adj2 (participat* or engag* or advisor* or partner* or involv* or collaborat* or lead or led or researcher*)).ti,ab,id. | 174 |
| 38 | ((user*-centred or user*-centered or user* oriented or patient*-centred or patient*-centered or patient* oriented or client-centered or client-centred or client* oriented or experience-based) adj3 (partnership* or research* or collaborat*)).ti,ab,id. | 782 |
| 39 | ((collaborat* or participatory) adj2 research*).ti,ab,id. and (patient* or client* or child* or youth or young people* or "service user*" or consumer* or survivor* or informant* or family or families or carer* or caregiver* or "lived expertise" or "living expertise" or "lived experience" or "living experience" or PWLE*).ti,ab,id,hw. | 5204 |
| 40 | or/25-39 | 21793 |
| 41 | 24 and 40 | 6900 |
| 42 | limit 41 to ("0800 literature review" or "0830 systematic review" or 1200 meta analysis or 1300 metasynthesis) | 416 |
| 43 | ((review* not book review*) or meta-analys* or metaanalys* or overview* or synthesis or metasynthesis).ti,id. | 243299 |
| 44 | (medline or embase or (psycinfo not psycinfo database record) or cinahl).ab. | 25251 |
| 45 | 41 and (43 or 44) | 410 |
| 46 | 42 or 45 | 574 |
| 47 | limit 46 to review-book | 53 |
| 48 | 46 not 47 | 521 |
